# Supplementary material for: Clostridioides difficile exploits toxin-mediated inflammation to alter the host nutritional landscape and exclude competitors from the gut microbiota
Source: Nat Commun. 2021 Jan 19;12:462. doi: 10.1038/s41467-020-20746-4 (PMC7815924; doi:10.1038/s41467-020-20746-4)
Supplement: Supplementary file 1 — Supplementary Information [file 41467_2020_20746_MOESM1_ESM.pdf]

Supplementary Information for *Clostridioides difficile* exploits toxin-mediated inflammation to alter the host nutritional landscape and exclude competitors from the gut microbiota

Authors: Joshua R. Fletcher[1], Colleen M. Pike[1], Ruth J. Parsons[1], Alissa J. Rivera[1], Matthew H. Foley[1], Michael R. McLaren[1], Stephanie A. Montgomery[2], Casey M. Theriot[1*]

Affiliations: [1]Department of Population Health and Pathobiology, College of Veterinary Medicine, North Carolina State University, 1060 William Moore Drive, Raleigh, NC 27607 [2]Department of Pathology and Laboratory Medicine, Lineberger Comprehensive Cancer Center, University of North Carolina School of Medicine, Chapel Hill, NC

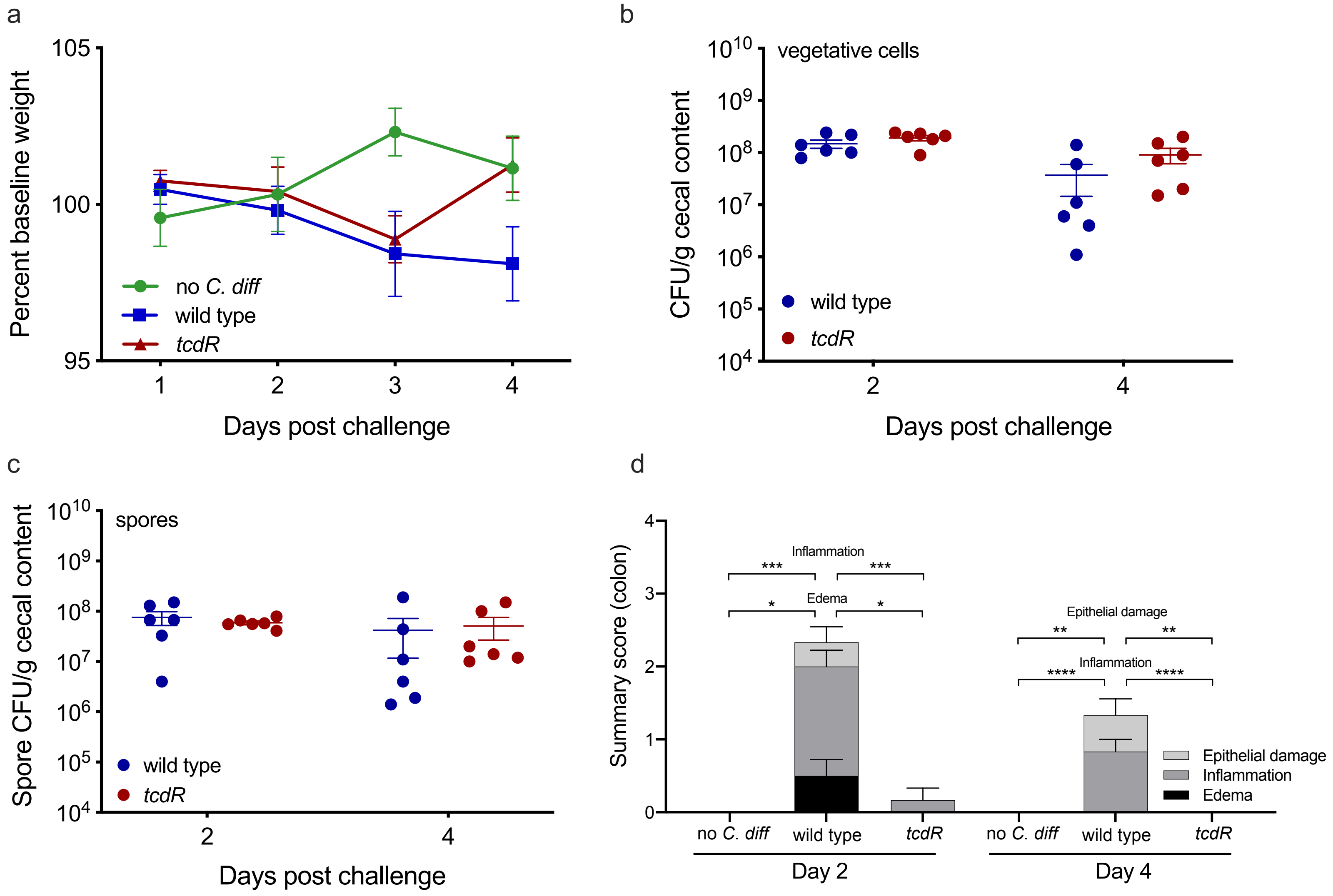

Supplementary Figure 1. Extended data from the mouse model of *C. difficile* infection. a) Mouse weights over the course of the experiments (n=12 mice on day 2, n=6 mice on day 4). b) Vegetative cell CFUs in the cecal content on day 2 and 4 (n=6 mice per group). c) Spore CFUs in cecal content from b (n=6 mice per group). d) Histopathological summary scores of the colon (n=6 mice per group per day). All data are presented as the mean and error bars indicate the SEM. **p=0.0012 and ****p<0.0001. Geissner-Greenhouse corrected ordinary Two-Way ANOVA with Tukey's multiple comparisons test was used to test for significance.

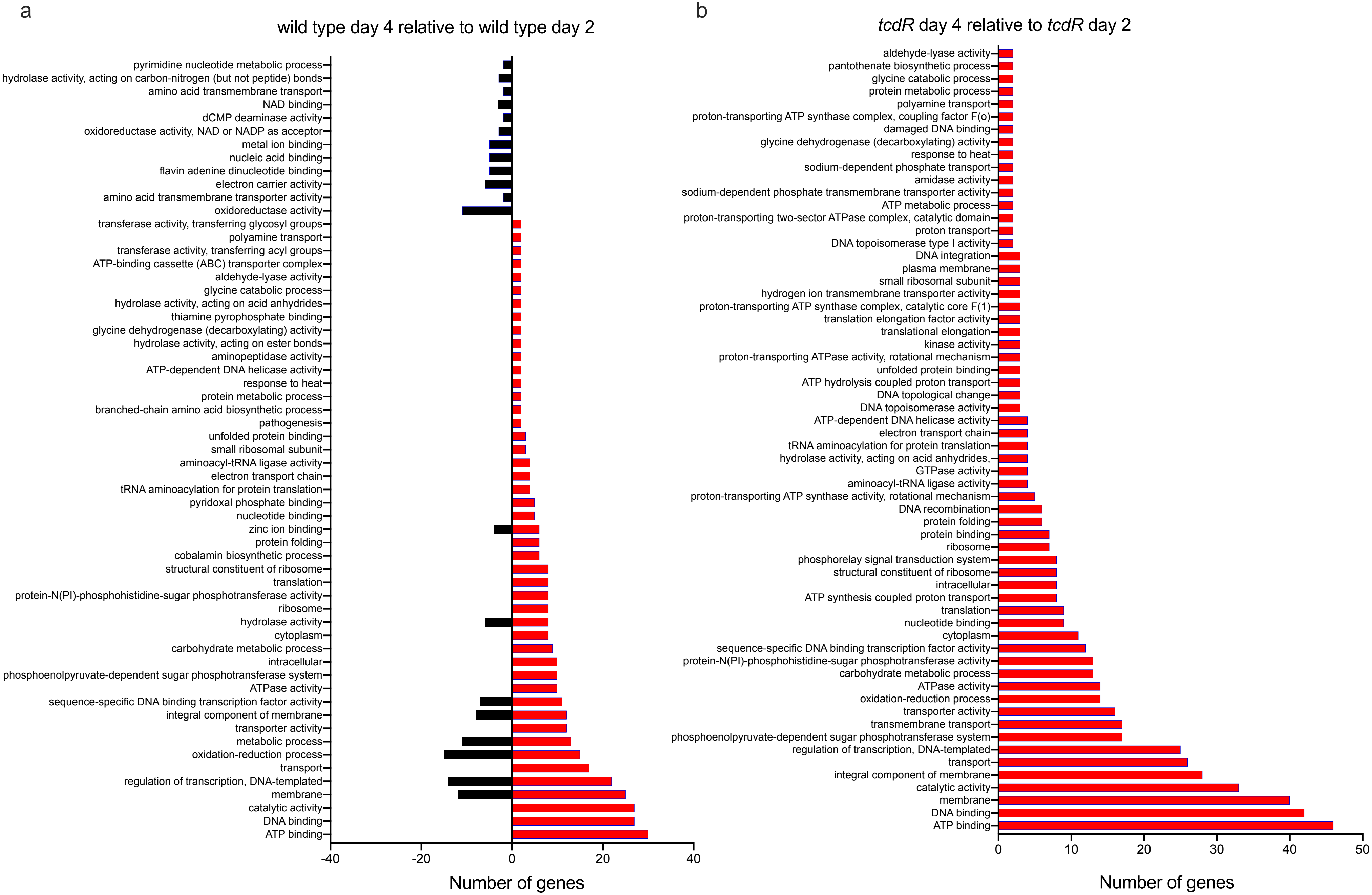

Supplementary Figure 2. *C. difficile* transcriptome shifts over the course of infection. a) Enriched GO terms in the differentially expressed genes in wild type *C. difficile* and b) *tcdR* at day 4 (n=3 mice per group) relative to day 2 (n=5-6 mice per group). GO terms for transcripts with decreased or increased levels are shown in black and red bars, respectively.

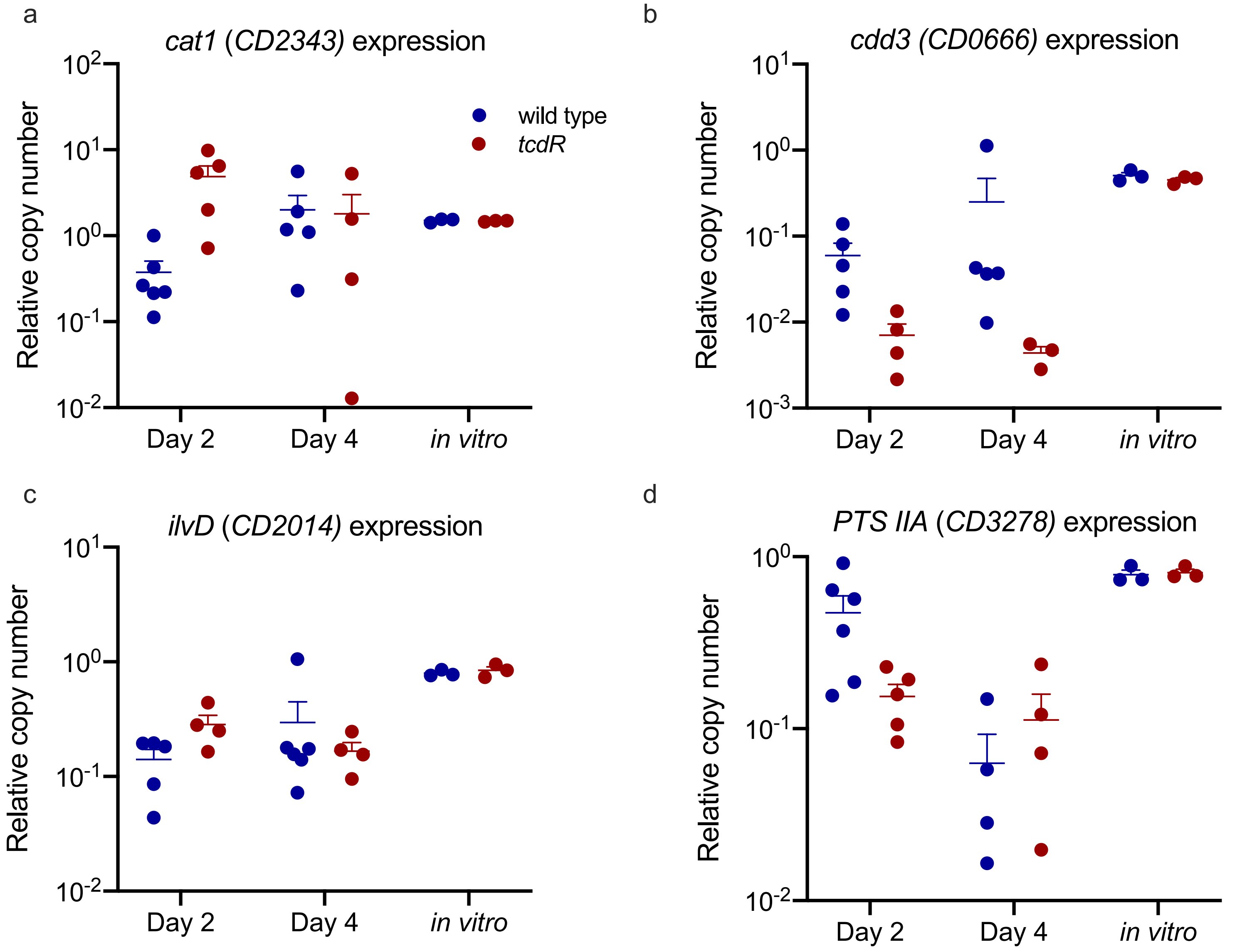

Supplementary Figure 3. Validation of differentially expressed *C. difficile* transcripts by qRT-PCR. a-d) Expression was quantified from cDNA generated from RNA isolated from cecal content and TY broth-grown *C. difficile* (in vitro). Each point represents a biological replicate. All data are presented as the mean and error bars indicate the SEM. Wild type n=5 mice at day 2, n=6 mice at day 4, n=3 in vitro; tcdR n=5 mice at day 2, n=5 mice at day 4, n=3 in vitro.

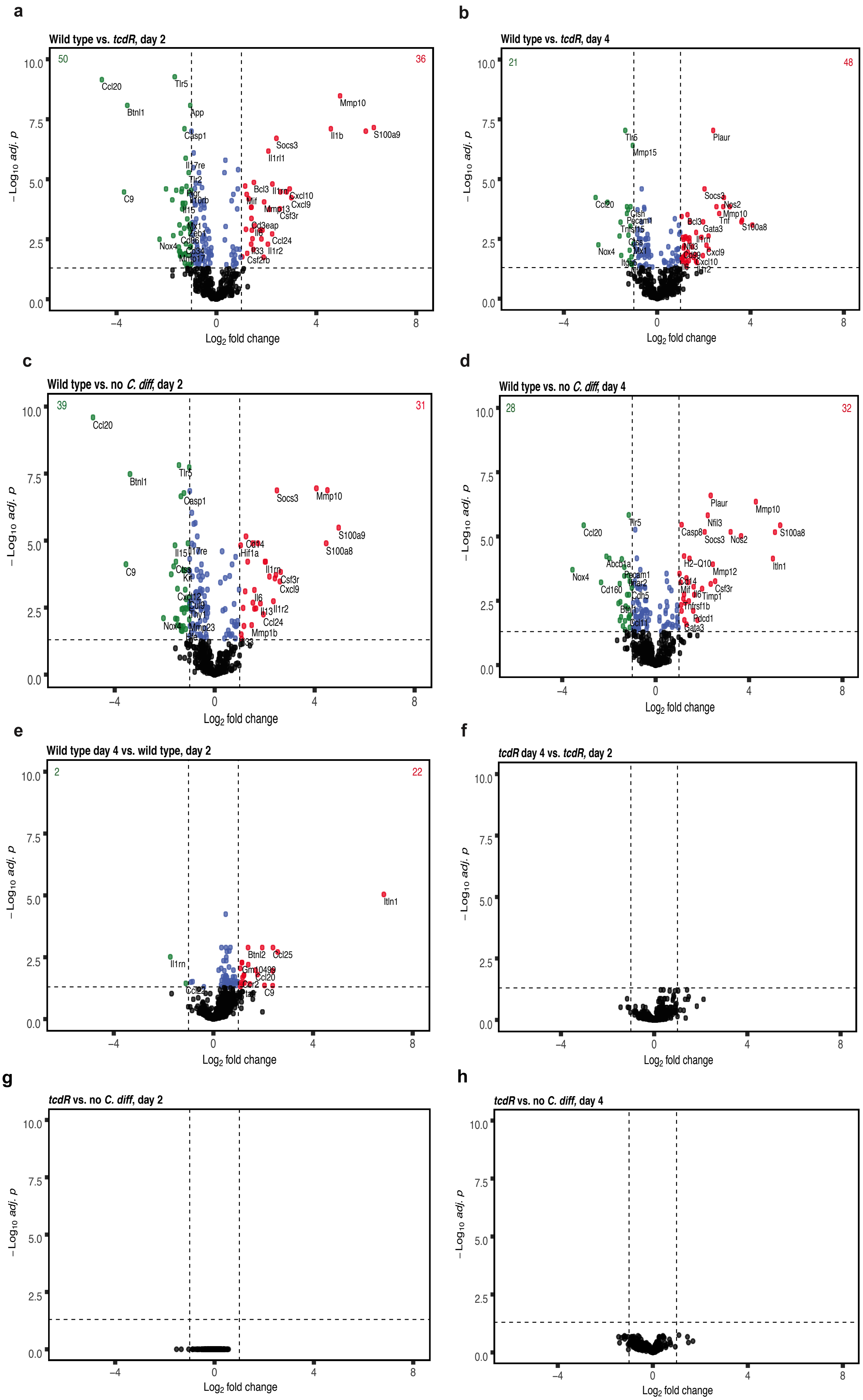

Supplementary Figure 4. Volcano plots depicting log2 fold change in expression and the adjusted p-value for mouse cecal tissue transcriptomes. a and b) Gene expression in wild type ceca relative to *tcdR* ceca at days 2 and 4, respectively. c and d) Gene expression in wild type ceca relative to ceca from uninfected controls (no *C. diff*) at 2 and 4 days, respectively. e and f) Gene expression at day 4 relative to day 2 in wild type ceca and *tcdR* ceca, respectively. The number of genes increased in expression are in red font and decreased are in green font.

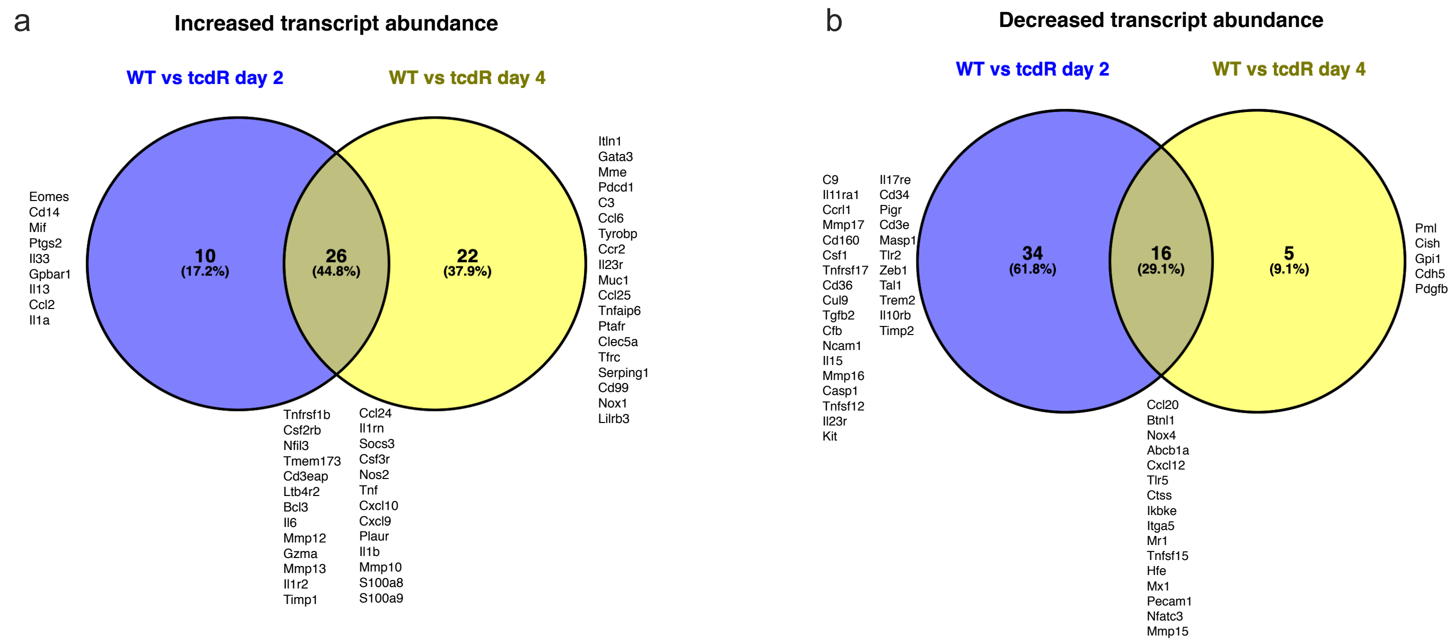

Supplementary Figure 5. Shared and unique differentially expressed genes in ceca of wild type relative to *tcdR* mice over time. a) Transcripts that increased in expression. b) Transcripts that decreased in expression. Venn diagrams were created using Venny 2.1 (https://bioinfogp.cnb.csic.es/tools/venny/).

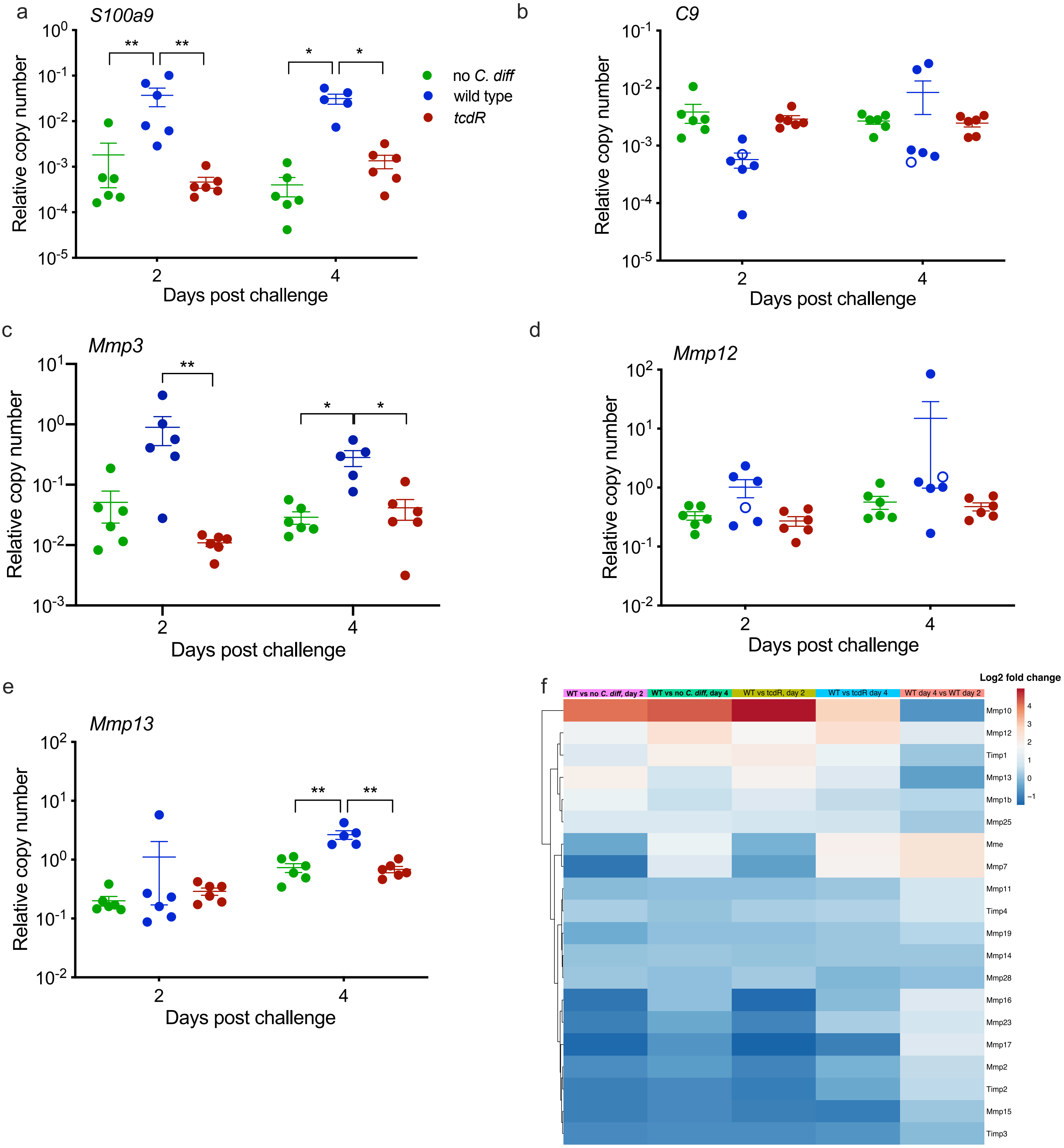

Supplementary Figure 6. Validation of transcripts from NanoString cecal tissue analysis by qRT-PCR. a-e) Expression was quantified in cDNA generated from the same cecal RNA that was used in the NanoString analysis (n=6 mice for all groups, except n=5 mice for wild type at day 4 in a, c, and e. For day 2 comparisons in a, **p=0.0053 for no *C. diff* vs. wild type, and **p=0.0035 for wild type vs. *tcdR*. For day 4, *p=0.0206 for no *C. diff* vs. wild type, and *p=0.0253 for wild type vs. *tcdR*. For day 2 comparisons in c, **p=0.0029 for wild type vs. *tcdR*. For day 4 comparisons in c, *p=0.0101 for no *C. diff* vs. wild type, and *p=0.0438 for wild type vs. *tcdR*. For day 4 comparisons in e, **p=0.0033 for no *C. diff* vs. wild type, and **p=0.0029. F) Unscaled heatmap depicting the log2 fold change of each *Mmp* and *Timp* in every differential expression analysis. The heatmap was created using the R package pheatmap (https://cran.r-project.org/web/packages/pheatmap/index.html). For Supplementary Figure 6b and d, a sample that we had identified as an outlier was inadvertently included in the qRT-PCR validation of *C9* and *Mmp12* expression levels. We have indicated the data point in each graph derived from this sample with an open circle. All data are presented as the mean and error bars indicate the SEM. A mixed effects model with Tukey's multiple comparison's test was used to test for significance.

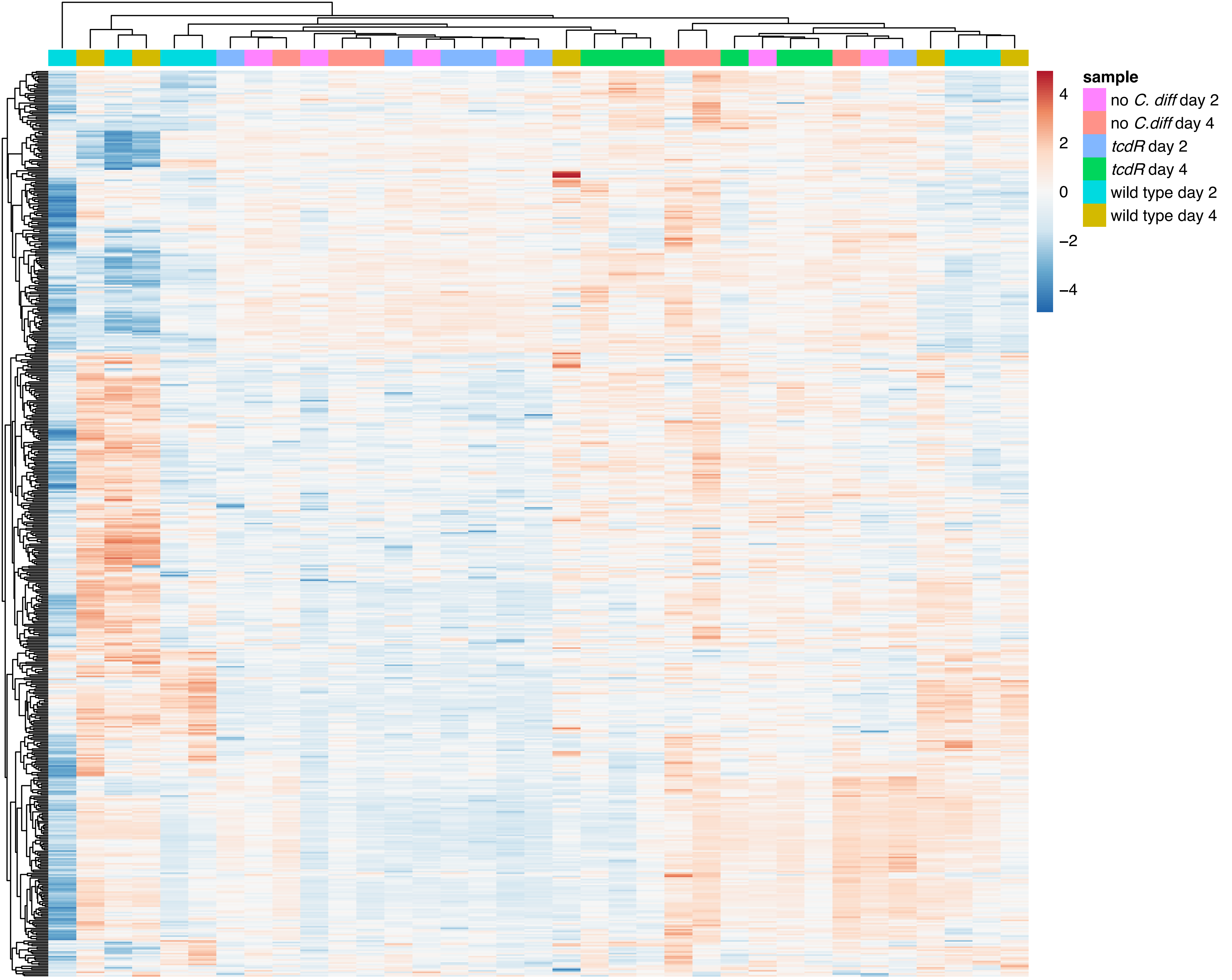

Supplementary Figure 7. Heatmap of all transcripts in the ceca of uninfected control (no *C. diff)*, wild type, and *tcdR* mice. (n=5-6 mice per group per day as seen in Fig. 3a).

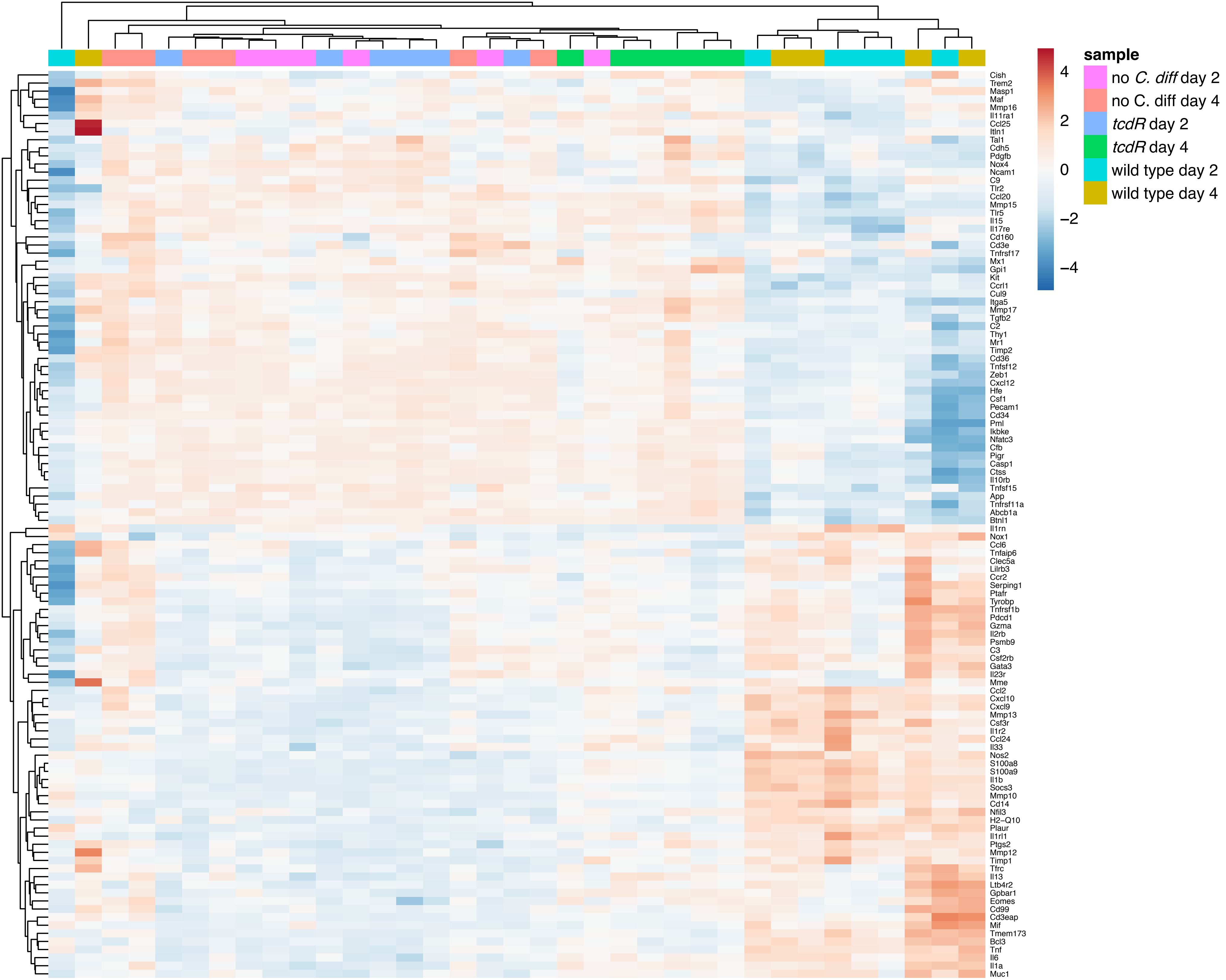

Supplementary Figure 8. Heatmap of all differentially regulated transcripts (by adj. p value) in the ceca of uninfected control (no *C. diff*), wild type, and *tcdR* mice. (n=5-6 mice per group per day as seen in Fig. 3a).
