# Supplementary material for: Clostridioides difficile exploits toxin-mediated inflammation to alter the host nutritional landscape and exclude competitors from the gut microbiota
Source: Nat Commun. 2021 Jan 19;12:462. doi: 10.1038/s41467-020-20746-4 (PMC7815924; doi:10.1038/s41467-020-20746-4)
Supplement: Supplementary file 3 — Description of Additional Supplementary Files [file 41467_2020_20746_MOESM3_ESM.docx]

**Description of Additional Supplementary Files**

File Name: Supplementary Data 1

Description: Lists of significant (adjusted p value < 0.05) differentially expressed transcripts between wild type *C. difficile* and the *tcdR* mutant *in vitro* and *in vivo*. Each sheet contains a different comparison.

File Name: Supplementary Data 2

Description: List of Gene Ontology (GO) terms from gene set enrichment analysis of significantly differentially expressed transcripts when comparing the transcriptomes of wild type *C. difficile* and the *tcdR* mutant *in vivo.*

File Name: Supplementary Data 3

Description: List of significant (adjusted p value < 0.05) differentially expressed transcripts in the cecal tissue of uninfected control mice, and mice infected with either wild type *C. difficile* or the *tcdR* mutant at two and four days post challenge.

File Name: Supplementary Data 4

Description: List of Gene Ontology (GO) terms from gene set enrichment analysis of significantly differentially expressed transcripts when comparing the cecal tissue transcriptomes of uninfected control mice, and mice infected with either wild type *C. difficile* or the *tcdR* mutant at two and four days after challenge.

File Name: Supplementary Data 5

Description: 16S rRNA ASVs detected in cecal tissue from uninfected control mice, and mice infected with wild type *C. difficile* or the *tcdR* mutant, at two and four days post challenge.

File Name: Supplementary Data 6

Description: 16S rRNA ASVs detected in cecal tissue from mice infected with wild type *C. difficile* R20291 and the isogenic ∆*tcdR* mutant four days post challenge.

File Name: Supplementary Data 7

Description: QIIME2 code used to analyze the cecal microbial community structures of uninfected control mice, and mice infected with wild type *C. difficile* or the *tcdR* mutant, at two and four days post challenge.

File Name: Supplementary Data 8

Description: QIIME2 code used to analyze the cecal microbial community structures of mice infected with wild type *C. difficile* R20291 and the isogenic ∆*tcdR* mutant four days post challenge.

File Name: Supplementary Data 9

Description: R code used to perform and visualize principal components analysis of the cecal microbiota from uninfected control mice, and mice infected with wild type *C. difficile* or the *tcdR* mutant, at two and four days post challenge.
