# Supplementary material for: Clostridioides difficile exploits toxin-mediated inflammation to alter the host nutritional landscape and exclude competitors from the gut microbiota
Source: Nat Commun. 2021 Jan 19;12:462. doi: 10.1038/s41467-020-20746-4 (PMC7815924; doi:10.1038/s41467-020-20746-4)
Supplement: Supplementary file 12 — Supplementary data 9 [file 41467_2020_20746_MOESM12_ESM.pdf]

Principal components analysis from QIIME2 profiles

Michael McLaren

2019-11-01

Contents

Setup . . . 1
Aggregate highly similar ASVs . . . 4
Make the plot . . . 5
Session info . . . 7

Setup

```
library(here)
#> here() starts at /home/michael/ncsu-drive/research/fletcher-foley
library(phyloseq)
library(biomformat)
library(tidyverse)
#> -- Attaching packages --------------------------------------- tidyverse 1.3.0 --
#> v ggplot2 3.3.2     v purrr   0.3.4
#> v tibble  3.0.1     v dplyr   1.0.0
#> v tidyr   1.1.0     v stringr 1.4.0
#> v readr   1.3.1     v forcats 0.5.0
#> -- Conflicts ------------------------------------------ tidyverse_conflicts() --
#> x dplyr::filter() masks stats::filter()
#> x dplyr::lag()    masks stats::lag()
library(vegan)
#> Loading required package: permute
#> Loading required package: lattice
#> This is vegan 2.5-6
library(cowplot) # Only needed for cowplot theme
#>
#> ********************************************************
#> Note: As of version 1.0.0, cowplot does not change the
#>   default ggplot2 theme anymore. To recover the previous
#>   behavior, execute:
#>   theme_set(theme_cowplot())
#> ********************************************************
library(ggrepel) # Only needed for taxa labels in PCA plot
library(janitor)
#>
#> Attaching package: 'janitor'
#> The following objects are masked from 'package:stats':
#>
#>     chisq.test, fisher.test
# Also used: Biostrings, broom, knitr
```

```r
theme_set(theme_cowplot(font_size = 12))
```

```r
# otu table -----------------------------------------------------------------
# Read in the biom file without extracting the entire qza file
qza_file <- here("data", "fletcher", "qiime2", "table.qza")
# create a new directory
tmp <- tempfile()
dir.create(tmp)
# Get the path to the fasta file within the qza object
flist <- unzip(qza_file, list = TRUE) %>%
    as_tibble
biom_file <- flist %>%
    filter(str_detect(Name, "\\.biom")) %>%
    pull(Name)
# unzip and load just the fasta file
unzip(qza_file, files = biom_file, exdir = tmp)
bm <- file.path(tmp, biom_file) %>%
    read_biom()
#> Warning in strsplit(conditionMessage(e), "\n"): input string 1 is invalid in
#> this locale
# Make otu table
otu <- biom_data(bm) %>%
    as("matrix") %>%
    otu_table(taxa_are_rows = TRUE)
dim(otu)
#> [1] 208  32
# reference sequences -------------------------------------------------------
# Read in the reference sequences files without extracting the entire qiime qza
qza_file <- here("data", "fletcher", "qiime2", "rep-seqs.qza")
# create a new directory
# tmp <- dir_create(file_temp())
tmp <- tempfile()
dir.create(tmp)
# Get the path to the fasta file within the qza object
flist <- unzip(qza_file, list = TRUE) %>%
    as_tibble
fasta_file <- flist %>%
    filter(str_detect(Name, "\\.fasta")) %>%
    pull(Name)
# unzip and load just the fasta file
unzip(qza_file, files = fasta_file, exdir = tmp)
rs <- file.path(tmp, fasta_file) %>%
    Biostrings::readDNAStringSet()
# sample metadata -----------------------------------------------------------
trts.fletcher <- c("cef", "wt", "tcdR")
cages.fletcher <- seq(200, 211)
sam <- tibble(sample = sample_names(otu)) %>%
    separate(sample, c("cage", "mouse", "day", "treatment"), sep = "-",
        remove = FALSE) %>%
    mutate(day = str_extract(day, "[0-9]+")) %>%
    mutate_at(vars(cage, mouse, day), as.integer) %>%
    mutate_at(vars(cage, mouse, day, treatment), as.factor) %>%
    mutate(treatment = fct_relevel(treatment, trts.fletcher)) %>%
```

```
    column_to_rownames("sample") %>%
    sample_data
head(sam)
#>              cage mouse day treatment
#> 200-2-d4-cef  200     2   4       cef
#> 200-3-d4-cef  200     3   4       cef
#> 201-1-d2-cef  201     1   2       cef
#> 201-2-d4-cef  201     2   4       cef
#> 201-3-d4-cef  201     3   4       cef
#> 202-1-d4-cef  202     1   4       cef
tail(sam)
#>               cage mouse day treatment
#> 209-3-d4-tcdR  209     3   4      tcdR
#> 210-1-d4-tcdR  210     1   4      tcdR
#> 210-2-d2-tcdR  210     2   2      tcdR
#> 210-3-d4-tcdR  210     3   4      tcdR
#> 211-1-d2-tcdR  211     1   2      tcdR
#> 211-3-d2-tcdR  211     3   2      tcdR
# taxonomy --------------------------------------------------------------------
qza_file <- here("data", "fletcher", "qiime2", "taxonomy.qza")
tmp <- tempfile()
dir.create(tmp)
flist <- unzip(qza_file, list = TRUE) %>%
    as_tibble
tax_file <- flist %>%
    filter(str_detect(Name, "taxonomy.tsv")) %>%
    pull(Name)
unzip(qza_file, files = tax_file, exdir = tmp)
# load and parse the taxonomy tsv file
rns <- c("kingdom", "phylum", "class", "order", "family", "genus", "species")
tax <- file.path(tmp, tax_file) %>%
    read_tsv(comment = "#") %>%
    clean_names %>%
    separate(taxon, rns, sep = ";", fill = "right") %>%
    mutate_at(rns, str_sub, 6) %>%
    select(-confidence) %>%
    column_to_rownames("feature_id") %>%
    as("matrix") %>%
    tax_table
#> Parsed with column specification:
#> cols(
#>   `Feature ID` = col_character(),
#>   Taxon = col_character(),
#>   Confidence = col_double()
#> )
# Make phyloseq object (with taxa as columns) ---------------------------------
ps <- phyloseq(otu, sam, rs, tax)
if (taxa_are_rows(ps))
    ps <- t(ps)
remove(otu, sam, rs, tax)
# Check in decreasing order
taxa_sums(ps) %>%
    all.equal(., sort(., decreasing = TRUE))
```

```r
#> [1] TRUE
# Simplify taxa names
taxa_names(ps) <- paste0("ASV", seq(ntaxa(ps)))
# save for future use
saveRDS(ps, here("results", "fletcher-qiime2-phyloseq.Rds"))
```

Aggregate highly similar ASVs

```r
d <- refseq(ps) %>%
    Biostrings::stringDist(method = "levenshtein")
Biostrings::width(refseq(ps)) %>% summary
#>    Min. 1st Qu.  Median    Mean 3rd Qu.    Max.
#>   252.0   253.0   253.0   252.9   253.0   254.0
# tree <- hclust(d) %>% as.phylo
hc <- hclust(d, method = "complete")
# plot(hc, hang=-1)
```

Form clusters where the distance between the furthest pair is $<$ 1%, or ~2.5, in an attempt to aggregate ASVs that are 99% similar.

```r
ct <- cutree(hc, h = 253*0.01) %>% enframe("taxon", "group")
# ct <- cutree(hc, h = 3) %>% enframe("taxon", "group")
archetypes <- taxa_sums(ps) %>%
    enframe("taxon", "sum") %>%
    left_join(ct, by = "taxon") %>%
    group_by(group) %>%
    top_n(1, sum) %>%
    slice(1) %>%
    select(group, archetype = taxon)
tb <- otu_table(ps) %>% as("matrix") %>% as_tibble(rownames = "sample") %>%
    gather("taxon", "abundance", -sample) %>%
    left_join(ct, by = "taxon") %>%
    group_by(sample, group) %>%
    summarize_at("abundance", sum) %>%
    left_join(archetypes, by = "group")
newotu <- tb %>%
    select(-group) %>%
    pivot_wider(names_from = archetype, values_from = abundance) %>%
    column_to_rownames("sample") %>%
    otu_table(taxa_are_rows = FALSE)
ps0 <- ps
otu_table(ps0) <- newotu
```

Check that the distances between groups are as expected. In particular, the minimum complete-linkage distance between any two groups should be 3.

```r
dist_groups <- function(d, g, diag = FALSE, upper = TRUE) {
    gtb <- tibble(item = labels(d) %>% factor(., .), group = factor(g))
    dtb <- d %>%
        broom::tidy(diag = diag, upper = upper) %>%
        left_join(gtb, by = c("item1" = "item")) %>%
        left_join(gtb, by = c("item2" = "item"), suffix = c("1", "2")) %>%
        select(-distance, distance)
}
```

```r
dtb <- dist_groups(d, ct$group)
# The complete-linkage dist between groups is the max dist between all pairs of
# items from the two groups; compute and then get the min dist between groups
dtb %>%
    group_by(group1, group2) %>%
    summarize_at("distance", max) %>%
    filter(group1 != group2) %>%
    pull(distance) %>%
    min
#> [1] 3
```

Check for tax concordance among the merged groups:

```r
tax <- tax_table(ps) %>% as("matrix") %>% as_tibble(rownames = "taxon")
# left_join(ct, archetypes, by = "group")
tb <- ct %>%
    left_join(archetypes, by = "group") %>%
    left_join(tax, by = "taxon") %>%
    unite(taxonomy, rns, sep = ";") %>%
    select(archetype, taxonomy) %>%
    group_by(archetype) %>%
    distinct %>%
    count
#> Note: Using an external vector in selections is ambiguous.
#> i Use `all_of(rns)` instead of `rns` to silence this message.
#> i See <https://tidyselect.r-lib.org/reference/faq-external-vector.html>.
#> This message is displayed once per session.
tb %>% pull(n) %>% summary
#>    Min. 1st Qu.  Median    Mean 3rd Qu.    Max.
#>   1.000   1.000   1.000   1.024   1.000   2.000
tb %>% filter(n > 1) %>%
    left_join(tax, by = c(archetype = "taxon"))
#> # A tibble: 4 x 9
#> # Groups:   archetype [4]
#>   archetype     n kingdom  phylum  class   order  family   genus      species
#>   <chr>     <int> <chr>    <chr>   <chr>   <chr>  <chr>    <chr>      <chr>
#> 1 ASV40         2 Bacteria Firmic~ Clostr~ Clost~ Lachnos~ Lachnospi~ <NA>
#> 2 ASV43         2 Bacteria Firmic~ Clostr~ Clost~ Lachnos~ Lachnoclo~ <NA>
#> 3 ASV66         2 Bacteria Firmic~ Clostr~ Clost~ Ruminoc~ uncultured <NA>
#> 4 ASV93         2 Bacteria Firmic~ Clostr~ Clost~ Lachnos~ Lachnospi~ unculture~
```

None of the taxa with non-concordant taxonony are labeled in the plot below, so we don't need to worry about adding NAs at their lower ranks.

Make the plot

```r
set.seed(43)
# ps0 <- ps
sam <- sample_data(ps0) %>% as("data.frame") %>% as_tibble(rownames = "sample")
tax <- tax_table(ps) %>% as("matrix") %>% as_tibble(rownames = "taxon")
# Hellinger PCA ----------------------------------------------------------------
ps0.hel.pca <- ps0 %>%
    transform_sample_counts(function(x) sqrt(x / sum(x))) %>%
    {rda(otu_table(.))}
```

```r
# Get a tibble for plotting
ps0.hel.pca.tb <- scores(ps0.hel.pca, 1:4, scaling = "sites") %>%
    # merge species and sites (samples) into a single dataframe
    map_dfr(as_tibble, rownames = "var", .id = "type") %>%
    # join with species and site metadata
    left_join(sam, by = c(var = "sample")) %>%
    left_join(tax, by = c(var = "taxon")) %>%
    mutate_at("type", fct_recode, taxon = "species", sample = "sites")
# percent variance explained, formatted for plotting
ps0.hel.pca.perc <- ps0.hel.pca %>% eigenvals %>% {. / sum(.)} %>%
    {round(100 * ., 0)}
# Data frame with taxonomy info for the labeled taxa. Pick top 10 taxa based on
# distance from the origin
tax.lab <- ps0.hel.pca.tb %>%
    filter(type == "taxon") %>%
    mutate(distance = sqrt(PC1^2 + PC2^2)) %>%
    top_n(10, distance) %>%
    select(taxon = var) %>%
    left_join(tax, by = "taxon") %>%
    arrange(str_sub(taxon, 4) %>% as.integer)
ps0.hel.pca.plt <- ps0.hel.pca.tb %>%
    mutate(PC2 = -PC2) %>%
    ggplot(aes(PC1, PC2)) +
    # axes
    geom_hline(yintercept = 0, color = "black") +
    geom_vline(xintercept = 0, color = "black") +
    # all taxa
    geom_point(data = ~filter(., type == "taxon"),
        aes(PC1/3, PC2/3),
        shape = 3, color = "darkgrey") +
    # samples
    geom_point(data = ~filter(., type == "sample"),
        aes(color = treatment, shape = day), size = 3) +
    # label subset of taxa
    geom_text_repel(data = ~filter(., var %in% tax.lab$taxon),
        aes(PC1/3, PC2/3, label = var), color = "black") +
    # geom_text_repel(data = ~filter(., type == "sample"), aes(label = cage)) +
    scale_color_brewer(type = "qual", palette = 2) +
    theme(axis.line = element_blank()) +
    labs(
        title = "PCA on Hellinger-transformed abundances",
        x = paste0("PC1 [", ps0.hel.pca.perc["PC1"], "%]"),
        y = paste0("PC2 [", ps0.hel.pca.perc["PC2"], "%]")
    )
```

```r
ps0.hel.pca.plt
```

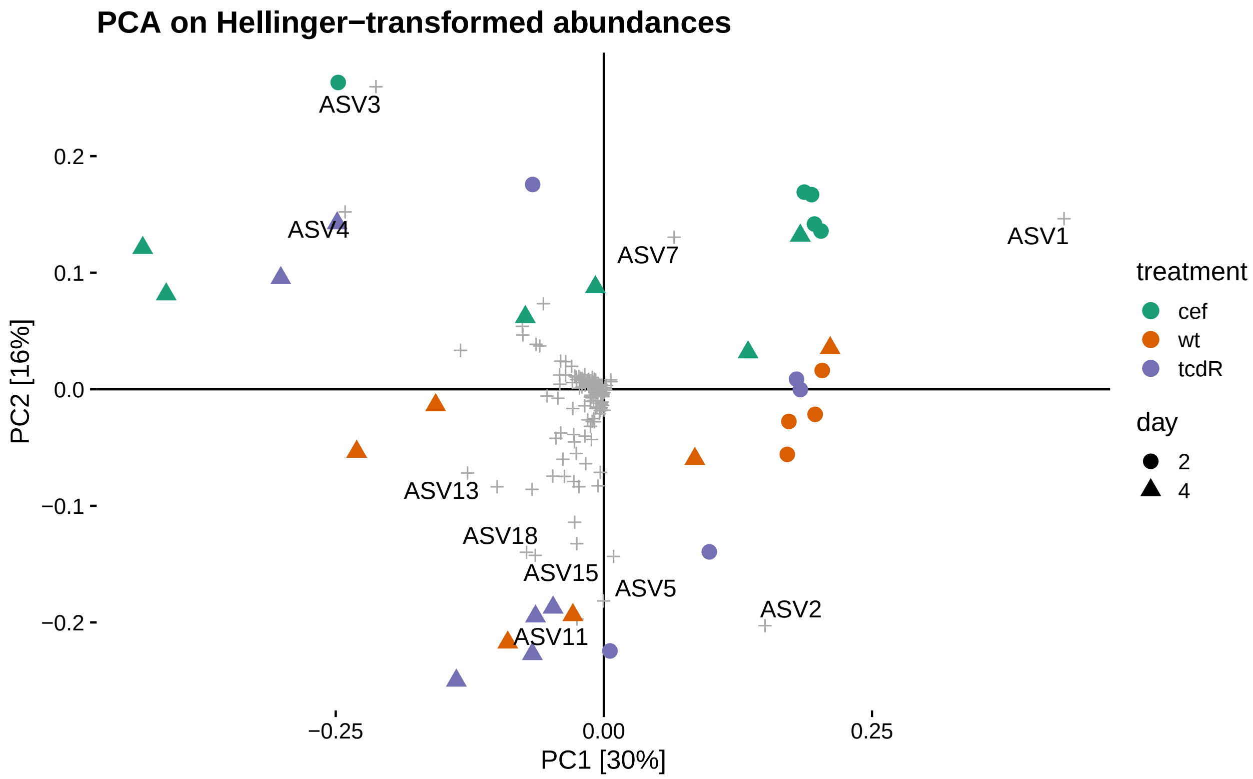


```r
ggsave(here("figures", "2019-11-5-qiime-microbiota-hellinger-pca.pdf"),
       useDingbats = FALSE, units = "in", width = 8, height = 5)
```

```r
tax.lab %>% knitr::kable()
```

taxon	kingdom	phylum	class	order	family	genus	species
ASV1	Bacteria	Firmicutes	Bacilli	Lactobacillales	Enterococcaceae	Enterococcus	NA
ASV2	Bacteria	Firmicutes	Clostridia	Clostridiales	Peptostreptococcaceae	Clostridioides	NA
ASV3	Bacteria	Bacteroidetes	Bacteroidia	Bacteroidales	Bacteroidaceae	Bacteroides	NA
ASV4	Bacteria	Verrucomicrobia	Verrucomicrobiae	Verrucomicrobiales	Akkermansiaceae	Akkermansia	NA
ASV5	Bacteria	Firmicutes	Clostridia	Clostridiales	Lachnospiraceae	ASF356	NA
ASV7	Bacteria	Firmicutes	Bacilli	Bacillales	Staphylococcaceae	Staphylococcus	NA
ASV11	Bacteria	Firmicutes	Clostridia	Clostridiales	Lachnospiraceae	NA	NA
ASV13	Bacteria	Firmicutes	Clostridia	Clostridiales	Lachnospiraceae	NA	NA
ASV15	Bacteria	Firmicutes	Erysipelotrichia	Erysipelotrichales	Erysipelotrichaceae	uncultured	uncultured bacterium
ASV18	Bacteria	Firmicutes	Clostridia	Clostridiales	Lachnospiraceae	NA	NA

```r
dna <- refseq(ps) %>% as.character %>% enframe("taxon", "sequence")
tax.lab %>%
    left_join(dna, by = "taxon") %>%
    write_csv(here("results", "2019-11-5-qiime-ordination-taxa.csv"))
```

Session info

```r
sessionInfo()
#> R version 4.0.2 (2020-06-22)
```

```
#> Platform: x86_64-pc-linux-gnu (64-bit)
#> Running under: Arch Linux
#>
#> Matrix products: default
#> BLAS:   /usr/lib/libblas.so.3.9.0
#> LAPACK: /usr/lib/liblapack.so.3.9.0
#>
#> locale:
#>  [1] LC_CTYPE=en_US.UTF-8       LC_NUMERIC=C
#>  [3] LC_TIME=en_US.UTF-8        LC_COLLATE=en_US.UTF-8
#>  [5] LC_MONETARY=en_US.UTF-8    LC_MESSAGES=en_US.UTF-8
#>  [7] LC_PAPER=en_US.UTF-8       LC_NAME=C
#>  [9] LC_ADDRESS=C               LC_TELEPHONE=C
#> [11] LC_MEASUREMENT=en_US.UTF-8 LC_IDENTIFICATION=C
#>
#> attached base packages:
#> [1] stats     graphics  grDevices utils     datasets  methods   base
#>
#> other attached packages:
#>  [1] janitor_2.0.1     ggrepel_0.8.2     cowplot_1.0.0     vegan_2.5-6
#>  [5] lattice_0.20-41   permute_0.9-5     forcats_0.5.0     stringr_1.4.0
#>  [9] dplyr_1.0.0       purrr_0.3.4       readr_1.3.1       tidyr_1.1.0
#> [13] tibble_3.0.1      ggplot2_3.3.2     tidyverse_1.3.0   biomformat_1.16.0
#> [17] phyloseq_1.32.0   here_0.1          rmarkdown_2.3     nvimcom_0.9-92
#> [21] usethis_1.6.1
#>
#> loaded via a namespace (and not attached):
#>  [1] nlme_3.1-148        fs_1.4.1            lubridate_1.7.9
#>  [4] RColorBrewer_1.1-2  httr_1.4.1          rprojroot_1.3-2
#>  [7] tools_4.0.2         backports_1.1.6     utf8_1.1.4
#> [10] R6_2.4.1            DBI_1.1.0           BiocGenerics_0.34.0
#> [13] mgcv_1.8-31         colorspace_1.4-1    ade4_1.7-15
#> [16] withr_2.2.0         tidyselect_1.1.0    compiler_4.0.2
#> [19] cli_2.0.2           rvest_0.3.5         Biobase_2.48.0
#> [22] xml2_1.3.2          labeling_0.3        scales_1.1.1
#> [25] digest_0.6.25       XVector_0.28.0      pkgconfig_2.0.3
#> [28] htmltools_0.5.0     highr_0.8           dbplyr_1.4.4
#> [31] rlang_0.4.6         readxl_1.3.1        rstudioapi_0.11
#> [34] farver_2.0.3        generics_0.0.2      jsonlite_1.7.0
#> [37] magrittr_1.5        Matrix_1.2-18       fansi_0.4.1
#> [40] Rcpp_1.0.4.6        munsell_0.5.0       S4Vectors_0.26.1
#> [43] Rhdf5lib_1.10.0     ape_5.4             lifecycle_0.2.0
#> [46] stringi_1.4.6       yaml_2.2.1          snakecase_0.11.0
#> [49] MASS_7.3-51.6       zlibbioc_1.34.0     rhdf5_2.32.1
#> [52] plyr_1.8.6          grid_4.0.2          blob_1.2.1
#> [55] parallel_4.0.2      crayon_1.3.4        Biostrings_2.56.0
#> [58] haven_2.3.1         splines_4.0.2       multtest_2.44.0
#> [61] hms_0.5.3           knitr_1.29          pillar_1.4.4
#> [64] igraph_1.2.5        reshape2_1.4.4      codetools_0.2-16
#> [67] stats4_4.0.2        reprex_0.3.0        glue_1.4.1
#> [70] evaluate_0.14       data.table_1.12.8   modelr_0.1.8
#> [73] vctrs_0.3.1         foreach_1.5.0       cellranger_1.1.0
#> [76] gtable_0.3.0        assertthat_0.2.1    xfun_0.15
```

```
#> [79] broom_0.5.6         survival_3.1-12     iterators_1.0.12
#> [82] IRanges_2.22.2      cluster_2.1.0       ellipsis_0.3.1
```
