# Supplementary material for: Clostridioides difficile exploits toxin-mediated inflammation to alter the host nutritional landscape and exclude competitors from the gut microbiota
Source: Nat Commun. 2021 Jan 19;12:462. doi: 10.1038/s41467-020-20746-4 (PMC7815924; doi:10.1038/s41467-020-20746-4)
Supplement: Supplementary file 13 — Reporting Summary [file 41467_2020_20746_MOESM13_ESM.pdf]

nature research

Corresponding author(s): *Double-blind peer review submissions: write DBPR and your manuscript number here instead of author names.*

Last updated by author(s): *YYYY-MM-DD*

Reporting Summary

Nature Research wishes to improve the reproducibility of the work that we publish. This form provides structure for consistency and transparency in reporting. For further information on Nature Research policies, see our Editorial Policies and the Editorial Policy Checklist.

Statistics

For all statistical analyses, confirm that the following items are present in the figure legend, table legend, main text, or Methods section.

n/a	Confirmed	
☐	☒	The exact sample size (*n*) for each experimental group/condition, given as a discrete number and unit of measurement
☐	☒	A statement on whether measurements were taken from distinct samples or whether the same sample was measured repeatedly
☐	☒	The statistical test(s) used AND whether they are one- or two-sided<br>*Only common tests should be described solely by name; describe more complex techniques in the Methods section.*
☐	☒	A description of all covariates tested
☐	☒	A description of any assumptions or corrections, such as tests of normality and adjustment for multiple comparisons
☐	☒	A full description of the statistical parameters including central tendency (e.g. means) or other basic estimates (e.g. regression coefficient) AND variation (e.g. standard deviation) or associated estimates of uncertainty (e.g. confidence intervals)
☐	☒	For null hypothesis testing, the test statistic (e.g. *F*, *t*, *r*) with confidence intervals, effect sizes, degrees of freedom and *P* value noted<br>*Give P values as exact values whenever suitable.*
☒	☐	For Bayesian analysis, information on the choice of priors and Markov chain Monte Carlo settings
☒	☐	For hierarchical and complex designs, identification of the appropriate level for tests and full reporting of outcomes
☒	☐	Estimates of effect sizes (e.g. Cohen's *d*, Pearson's *r*), indicating how they were calculated

Our web collection on statistics for biologists contains articles on many of the points above.

Software and code

Policy information about availability of computer code

Data collection	no software was used.
Data analysis	The 16S rRNA sequence data was analyzed in Qiime2 and the code is available in Supplemental Files 7-8. The R code can be found in Supplemental File 9. The essential R packages used were biomformat, Biostrings, phyloseq, vegan, ggplot2, and data importing and manipulation packages from the tidyverse package collection.

For manuscripts utilizing custom algorithms or software that are central to the research but not yet described in published literature, software must be made available to editors and reviewers. We strongly encourage code deposition in a community repository (e.g. GitHub). See the Nature Research guidelines for submitting code & software for further information.

Data

Policy information about availability of data

All manuscripts must include a data availability statement. This statement should provide the following information, where applicable:

- Accession codes, unique identifiers, or web links for publicly available datasets
- A list of figures that have associated raw data
- A description of any restrictions on data availability

Raw sequences have been deposited in the Sequence Read Archive (SRA) with SRA accession number SUB6663505 and BioProject ID PRJNA612095. Additioanal raw data is provided in Supplemental Files.

Field-specific reporting

Please select the one below that is the best fit for your research. If you are not sure, read the appropriate sections before making your selection.

- [x] Life sciences
- [ ] Behavioural & social sciences
- [ ] Ecological, evolutionary & environmental sciences

For a reference copy of the document with all sections, see nature.com/documents/nr-reporting-summary-flat.pdf

Life sciences study design

All studies must disclose on these points even when the disclosure is negative.

Sample size	Statistical analysis of variance (parametric and non-parametric statistical test) were used to compare the different groups of mice across the studies based on the following parameters: weight loss, bacterial load, toxin activity, and histopathological parameters, and omics data. The number of mice used in this study was n=6-8 per group (male and female) and was necessary for statistical power and to control for cage-to-cage variation and gender differences. See methods and all figure legends for more details.
Data exclusions	Nanostring expression data from one mouse was excluded as visual inspection of heat map and principal coordinates analysis data indicated that it was an outlier.
Replication	Attempts at replication were successful. See similar data on two completely different C. difficile strains used in this study.
Randomization	Mice were randomly selected for necropsy from different cages on day 2 and day 4 post C. difficile challenge. This should account for cage to cage variation.
Blinding	We were not blinded to the groups of mice in this study, but there were no differences in clinical signs of disease in the mice during this time. It was only through down stream analysis were we able to see a differences in the C. difficile load, toxin activity, and omics analysis. The tissue samples were blinded and randomized when being scored by a board certified pathologist (see methods section for more details).

Reporting for specific materials, systems and methods

We require information from authors about some types of materials, experimental systems and methods used in many studies. Here, indicate whether each material, system or method listed is relevant to your study. If you are not sure if a list item applies to your research, read the appropriate section before selecting a response.

Materials & experimental systems

n/a	Involved in the study	
[ ]	[x]	Antibodies
[ ]	[x]	Eukaryotic cell lines
[x]	[ ]	Palaeontology and archaeology
[ ]	[x]	Animals and other organisms
[x]	[ ]	Human research participants
[x]	[ ]	Clinical data
[x]	[ ]	Dual use research of concern

Methods

n/a	Involved in the study	
[x]	[ ]	ChIP-seq
[x]	[ ]	Flow cytometry
[x]	[ ]	MRI-based neuroimaging

Antibodies

Antibodies used	Goat anti-Mouse IgG, Alexa Fluor 488, ThermoFisher, A-11029, lot#2124366; COL5A1, Santa Cruz, sc-166155, mouse monoclonal IgG2a, lot#F1818; COL3A1, Santa Cruz, sc-271249, mouse monoclonal IgG1, lot#K2719; COL1A, Santa Cruz, sc-59772, mouse monoclonal IgG1, lot#I2719
Validation	COL5A1 has been validated by the manufacturer and previous publications:<br>https://datasheets.scbt.com/sc-166155.pdf<br>1. Berchtold, S., et al. 2015. Collagen type V promotes the malignant phenotype of pancreatic ductal adenocarcinoma. Cancer Lett. 356: 721-732.<br>2. Chen, J., et al. 2017. Substance P and patterned silk biomaterial stimulate periodontal ligament stem cells to form corneal stroma in a bioengineered three-dimensional model. Stem Cell Res. Ther. 8: 260.<br>3. Zhang, W., et al. 2017. Surface topography and mechanical strain promote keratocyte phenotype and extracellular matrix formation in a biomimetic 3D corneal model. Adv. Healthc. Mater. E-published.<br><br>COL3A1 has been validated by the manufacturer and previous publications:<br>https://datasheets.scbt.com/sc-271249.pdf<br>1. Ferro, F., et al. 2011. Adipose tissue-derived stem cell in vitro differentiation in a three-dimensional dental bud structure. Am. J. Pathol. 178: 2299-2310.

2. Rosell-Garcia, T. and Rodriguez-Pascual, F. 2018. Enhancement of collagen deposition and cross-linking by coupling lysyl oxidase with bone morphogenetic protein-1 and its application in tissue engineering. Sci. Rep. 8: 10780.
3. Li, L., et al. 2018. Microtubule associated protein 4 phosphorylation leads to pathological cardiac remodeling in mice. EBioMedicine 37: 221-235.

COL1A has been validated by the manufacturer and previous publications:
https://datasheets.scbt.com/sc-59772.pdf
1. Dooley, S., et al. 2008. Hepatocyte-specific Smad7 expression attenuates TGF-b-mediated fibrogenesis and protects against liver damage. Gastroenterology 135: 642-659.
2. El-Domyati, M., et al. 2015. Microneedling therapy for atrophic acne scars: an objective evaluation. J. Clin. Aesthet. Dermatol. 8: 36-42.
3. Ekizer, A., et al. 2015. Bone marrow mesenchymal stem cells enhance bone formation in orthodontically expanded maxillae in rats. Angle Orthod. 85: 394-399.

Eukaryotic cell lines

Policy information about cell lines

Cell line source(s)	Vero cells and IMR90 cells
Authentication	Cell lines were not authenticated.
Mycoplasma contamination	Cell lines were not tested for mycoplasma.
Commonly misidentified lines (See ICLAC register)	*Name any commonly misidentified cell lines used in the study and provide a rationale for their use.*

Animals and other organisms

Policy information about studies involving animals; ARRIVE guidelines recommended for reporting animal research

Laboratory animals	C57BL/6J WT mice (5 to 8 weeks old; n=18 male and n=18 female) were purchased from Jackson Labs.
Wild animals	This study did not involve wild animals.
Field-collected samples	This study did not involve samples collected from the field.
Ethics oversight	Please note that this information needs to be added to the manuscript: Male and female C57BL/6J mice (aged 5 weeks old) were purchased from Jackson Labs (Bar Harbor, ME) for use in infection experiments. The food, bedding, and water were autoclaved, and all cage changes were performed in a laminar flow hood. The mice were subjected to a 12 hr light and 12 hr dark cycle. Animal experiments were conducted in the Laboratory Animal Facilities located on the NCSU CVM campus. The animal facilities are equipped with a full time animal care staff coordinated by the Laboratory Animal Resources (LAR) division at NCSU. The NCSU CVM is accredited by the Association for the Assessment and Accreditation of Laboratory Animal Care International (AAALAC). Trained animal handlers in the facility fed and assessed the status of animals several times per day. Those assessed as moribund were humanely euthanized by CO2 asphyxiation.

Note that full information on the approval of the study protocol must also be provided in the manuscript.
